# Loss of MiR-155 Sensitizes FLT3-ITD^+^AML to Chemotherapy and FLT3 Inhibitors via Glycolysis Blocking by Targeting PIK3R1

**DOI:** 10.7150/jca.54775

**Published:** 2023-01-01

**Authors:** Lingyan Wang, Peifang Jiang, Jiazheng Li, Yan Huang, Jingjing Wen, Zhengjun Wu, Yanxin Chen, Jianda Hu

**Affiliations:** Fujian Medical University Union Hospital, Fujian Provincial Key Laboratory on Hematology, Fujian Institute of Hematology, Fuzhou 350001

**Keywords:** Aerobic Glycolysis, MiR-155, FLT3-ITD, Quizartinib (AC220), Drug Resistance

## Abstract

FLT3 tyrosine kinase inhibitors in combination with chemotherapy have shown some success in patients with FLT3 mutations. But a variety of mechanisms have led to the rapid resistance to the treatment. One of the most prominent is the metabolic alteration on aerobic glycolysis. We aim to explore the role of a high expressing microRNA, miR-155, in mediating resistance to chemotherapy and FLT3 inhibitor treatment. The deep sequencing data mining revealed the connection between glycolysis and drug resistance. MV411 cells with miR-155 knockout (KO) not only had increased sensitivity to FLT3 inhibitors but also Adriamycin (ADM) treatment. When combined with glycolysis inhibition the treatment response in MV411 cells further increased. Whereas in miR-155 KO cells, a lower glucose consumption level and lactic acid level were observed, and western blotting showed a decreased expression of key enzymes in glycolysis pathways. A negative correlation between PIK3R1 and miR-155 level can be observed in the sequencing data from FLT3-ITD^+^ AML patients. Moreover, luciferase reporter assay revealed that the 3'UTR of PIK3R1 mRNA can interact with the seed sequence of miR-155-5p. In conclusion, the loss of miR-155 increased treatment sensitivity to both chemotherapy and FLT3 inhibitors in FLT3-ITD^+^ AML cells via glycolysis blocking by targeting PIK3R1.

## Introduction

The FMS-like tyrosine kinase-3 internal tandem duplication (FLT3-ITD) is one of the most frequent mutations found in patients with acute myeloid leukemia (AML) and has been linked to a variety of other cancers including lung, thyroid, colorectal and breast carcinomas 1, 2. Its upregulation is correlated with an overall poor disease prognosis, and as such FLT3-ITD represents a driver lesion and valid drug therapy target 3-6. Inhibiting FLT3-ITD by using small molecules to interfere with FLT3's kinase activity represents one promising approach. Tyrosine kinase inhibitors (TKIs) work via competitive inhibition with ATP, binding to the active pockets of the kinases instead and resulting in the inability to auto-phosphorylate or phosphorylate substrate proteins 7, 8. Although no FLT3 inhibitors have as yet been approved for clinical use, several are currently under clinical trials for the treatment of relapsed or refractory FLT3-ITD positive and negative AML patients as well as for maintenance therapy 9-11. Unfortunately, even at this early stage a variety of resistance mechanisms for FLT3 inhibitors have been reported, with several studies identifying the mutations in FLT3 as conferring the drug resistance 12, 13.

Quizartinib (AC220) is a once-daily, orally administered, potent and selective second-generation FLT3 inhibitor for the treatment of AML 14. It has been shown to have very selective and sensitive *in vitro* and *in vivo* activity against FLT3. Compared to other FLT3 inhibitors, AC220 appears to be 1-2 orders of magnitude more potent *in vivo* 15. As such, AC220-resistant FLT3 kinase domain mutants represent high-value targets for future FLT3 inhibitor development. However, these efforts are stymied as the drug resistant mutations in the downstream pathways of FLT3 have yet to be systematically studied.

MicroRNA-155 (miR-155) is a well-known oncogenic microRNA, upregulated in FLT3-ITD^+^ AML. High expression of miR-155 independently predicts poor outcomes in cytogenetically normal patients and is associated with high risk FLT3-ITD mutations 16, 17. The Warburg effect is a common feature of cancer cells and hallmark of tumorigenesis, characterized by an abnormal metabolic shift to increased aerobic glycolysis. This metabolic shift is observed in leukemia and understanding how cancer cells drive such metabolic shifts is crucial to identify potential targets for cancer therapeutics 18, 19. Recent studies have proven that miR-155 plays a role in regulating metabolism, demonstrating that miR-155 upregulates HK2 through the activation of STAT3, while the suppression of miR-143 targets HK2 20. Moreover, studies have shown that miR-155 positively regulates glucose metabolism via the PIK3R1-FOXO3a-cMYC axis in breast cancer 21.

Herein, we demonstrate a correlation in FLT3-ITD^+^ cells between drug resistance and metabolism, and miR-155 levels. We elucidated miR-155's function in drug resistance through the modulation of glucose metabolism. We generated the miR-155 KO cell line model via the CRISPR/Cas9 tool and using this cell model, investigated glucose metabolism levels and TKI sensitivity, finding miR-155 to have a positive modulating role on both. By inhibiting glycolysis, we produced an enhanced anti-leukemia TKI effect. Moreover, we found that miR-155 modulates glycolysis through PI3KR1 mediated signaling. Our study provides an understanding of the mechanisms of resistance to TKIs through metabolic alterations and illuminates new potential therapeutic strategies in the treatment of FLT3-ITD leukemia, suggesting that glycolytic inhibition may override drug resistance.

## Materials and methods

### Cell culture

The MV411 cells were obtained from American Type Culture Collection (ATCC) and authenticated. The MV411 cells were cultured in Iscove's Modified Dulbecco's Medium (IMDM, Hyclone) supplemented with 10% fetal bovine serum (FBS, gibico) and 1% penicillin/streptomycin (Hyclone).

### Generation miR-155 knockout MV411 cells by CRISPR/Cas9

We used CRISPR/Cas9 system to knockout miR-155 genome. Two sgRNAs targeting miR-155 were designed from the website https://portals.broadinstitute.org/gpp/public/analysis-tools/sgrna-design.

The targeting sequences were as following:

sgRNA1(PCA06825): GTTAATGCTAATCGTGATAG

sgRNA2 (PCA06826): CACTGTTAATGCTAATATGT

nontargeting control (CON250): CGCTTCCGCGGCCCGTTCAA

MV411 cells carrying sgRNA1 were labeled as KO25, the ones with sgRNA2 were labeled as KO26, and the non-targeting controls, which are the vector with scramble sequence, were labeled as SCR. The vector construction and lentivirus production were performed by Shanghai Genechem Co., LTD. In brief, the sgRNA was cloned into the vector GV392 (U6-sgRNA-EF1a-Cas9-FLAG-P2A-puro). The ligated products were then transformed into the TOP10 competent cells, and the positive clones were validated by PCR reaction and sanger sequencing to obtain the lentivirus plasmids with correct insertion. Then the lentivirus plasmids as well as two other package plasmids carrying CMV and VSV-g were added to cultured HEK293T cells to package the lentiviral particles.

For transduction, MV411 cells were incubated in a 96-well plate with a density of 5,000 cells/well in 100 μL of culture medium and cultured to a confluency of 70%-80%, before the viral suspensions were added to each well and thoroughly mixed, followed by a 48-hour incubation. Next, we performed a surveyor assay and sanger sequencing to validate the miR-155 knock out cells.

### Mutagenesis determination

We used a surveyor assay to determine the mutation generated by CRISPR/Cas9. Briefly, genomic DNA was extracted from cells using Quickextract kit (Lucigen). The targeted locus was amplified by PCR for 30 cycles using following two primers:

Forward: CCTGAACCCTACCCTTATAAAACAGG

Reverse: AAAACGTTGCCAGACAATCC

The PCR products were heated at 95 °C for 10 min and cooled to 25°C with a 0.3°C drop per second to generate mismatched DNA duplex. Surveyor Nuclease (IDT) was added and the digested sample was resolved on a 2% agarose gel. The DNA bands were quantified using ImageJ software and the mutation rate in a cell population was calculated as mentioned in the literature 22.

### Overexpression of miR-155-5p

For the overexpression of miR-155-5p, we used a lentiviral vector carrying green fluorescence protein (GFP) to encode miR-155-5p (miR-155 OE). The lentivirus (miR-155 OE and control ONC) was produced as described above for miR-155 KO. For the miRNA overexpression, MV411 cells were transduced with lentivirus carrying miR-155 OE for 48 hours and validated by qPCR.

### Cell viability evaluation

We used MTT to make growth curve and the clone genic assay to evaluate the cell growth ability, and the methods were described in previous publication 23.

### Drug sensitivity detection

MV411 cells were seeded with 3000 cells/100 μL/well in triplicate in 96-well plates with a drug concentration ranging from 0 to 20 µM. Before detection, 20 μL of 5 mg/mL MTT was added to each well. After further incubation for 4 hours, the supernatant was removed and replaced with 100 μL DMSO in each well. The plates were then shaken on an orbital shaker for 10 min until formazan crystals were solubilized. The absorbance values (OD value) were then measured with dual wavelengths of 480 nm and 630 nm on a microplate reader (Bio-Tek, US). The assays were repeated three times.

### Drug accumulation detection

Intracellular ADM accumulation was also evaluated by flow cytometry (BD Biosciences). MV411 cells with miR-155 KO or SCR were cultured with 0.1 µg/ml ADM for 24 hours, then collected. The ADM median fluorescence intensity (MFI) was determined by the flow cytometer X (BD Biosciences) and the results were analyzed using flowjo Version X.

### Glucose consumption rate

10^5^ MV411 and other cultured cells were seeded in 12-well plates for glucose and lactic acid measurement. We used Glucose oxidase assay kit (Jianchen, Nanjing) to measure the glucose remaining in the media. To measure the glucose consumption rate, we collected the supernatant of cultured cells above and measured the start and stop time points of glucose remaining in the media and calculated a relative glucose consumption rate.

### Lactate generation measurement

Intracellular and extracellular lactate content of MV411 cells was determined using a Lactate Assay Kit (Biotime, Shanghai). Cells were cultured in the IMDM basic medium supplemented with 10% FBS. The cell pellet and culture medium were collected for lactate content measurement according to the manufacturer's instructions.

### ATP measurement

The level of intracellular ATP was measured by the Luminescent ATP Detection Assay Kit (Biotime, Shanghai). 1 × 10^6^ cells/mL were seeded in triplicate in six well plates and treated with the indicated compounds. After normalization, 100 μL of cells was transferred to a 96-well plate and mixed with 100 μL of reagents. The mixture was incubated at room temperature for 10 min before luminescence was detected by a microplate reader (Bioteck).

### Reporter construction and luciferase assay

For reporter construction, reverse complement oligonucleotide for matured miR-155 sequence was introduced into the pSI-Check2 Luciferase vector (Hanbio Biotechnology, China) via XhoI and NotI^24^ sites. Cells were transfected with 2 µg of plasmid DNA, which included either the control vector or the miR-155 3'-UTR Luciferase reporter vector, using Lipofectamine2000 (Invitrogen) to generate the vector carrying wild type or mutant (PIK3R1-3UTR-WT or PIK3R1-3UTR-MUT). 48 hours after transfection, cells were lysed and luciferase activity was measured using Dual-Luciferase Reporter Assay System (Promega Corp, Madison, WI, USA) and a Victor Luminometer (Perkin-Elmer, Waltham, MA, USA) according to the manufacturer's protocol.

### Quantitative RT PCR

We used the trizol reagent (invitrogen) to extract RNA and the miScript RT Kit II (Qiagen) for reverse transcription. The quantified PCR was performed with 1 µL cDNA using the miScript SYBR Green PCR Kit (Qiagen) and appropriate primers (Qiagen). The RNU6B was used as an internal control. The relative expression levels were calculated by 2^-ΔΔCt^ and all the reactions were done in parallel triplicate.

### Western blot

The total protein was extracted using RIPA buffer (Thermo Fisher Scientific) added to the cocktail of proteinase inhibitor and phosphorylase inhibitor. The Western blot procedure was performed as has been done previously 15. The primary antibodies: Phosphofructokinase (PFK), Platelet-type phosphofructokinase (PFKP), GAPDH, the M1 and M2 isoform of Pyruvate kinase (PKM1 and PKM2) and LDHA were from Glycolysis Antibody Sampler Kit #8337 (cell signaling technology, CST). For secondary antibodies either anti-rabbit (#14708) or anti-mouse (#14709) IgG conjugated with horseradish peroxidase (CST) were used. Western Lightning ECL (Bio-Red) reagents were used for fluorescence production and a ChemiDoc™ Imaging System (Bio-Rad Laboratories, US) was used to visualize the protein detected.

### Bioinformatic Data Processing

The RNA sequencing data of sorafenib resistant MV411 cells were download from Gene Expression Omnibus (GEO) dataset GSE74666. The RNA sequencing data of miR-155 knockdown MV411 cells were obtained from GSE71541. The CRISPR whole gene knockout sequencing data were from GSE105161. To analyze the AML patient's data, we download the RNA sequencing data from The Cancer Genome Atlas (TCGA). We also obtained the RNA and miRNA microarray data from European Bioinformatics Institute (EMBL-EBI https://www.ebi.ac.uk/arrayexpress/) with the accession numbers E-TABM-1032 and E-TABM-1029.

The miRNA-target interactions were predicted by an open-source platform, Encyclopedia of RNA Interactomes (ENCORI http://starbase.sysu.edu.cn/ version 3.0), which established eight miRNA-target prediction databases including PITA, RNA22, miRmap, microT, miRanda, PicTar, TargetScan, and pancancerNum.

Multiple geneset, function, and pathways enrichment was done using the online tool Enrichr (https://amp.pharm.mssm.edu/Enrichr/) 25, 26 and Metascape (https://metascape.org/) 27. Gene Set Enrichment Analysis (GSEA) was performed following the instructions of https://www.gsea-msigdb.org/gsea/ 28, 29.

### Statistical analysis

Data are presented as standard error of mean (SEM). For the comparisons of two cohorts, statistical significance was determined by the two tailed Student's T-test. One-way ANOVA was used to analyze the remaining data. Non-linear regression and Pearson correlation were used for correlation analysis, and the goodness of fit were evaluated by r square. p-value less than 0.05 was regarded as statistically significant. For the whole text, * stands for p<0.05, ** stands for p<0.01, *** stands for p<0.001, **** stands for p<0.0001.

## Results

### Data mining show the connection between drug sensitivity and aerobic glycolysis

Our results provide several pieces of evidence highlighting the connection between drug sensitivity and glucose metabolism. Various bioinformatic analysis revealed that FLT3 inhibitors' sensitivity is correlated with aerobic glycolysis related pathways.

**Figure 1:**
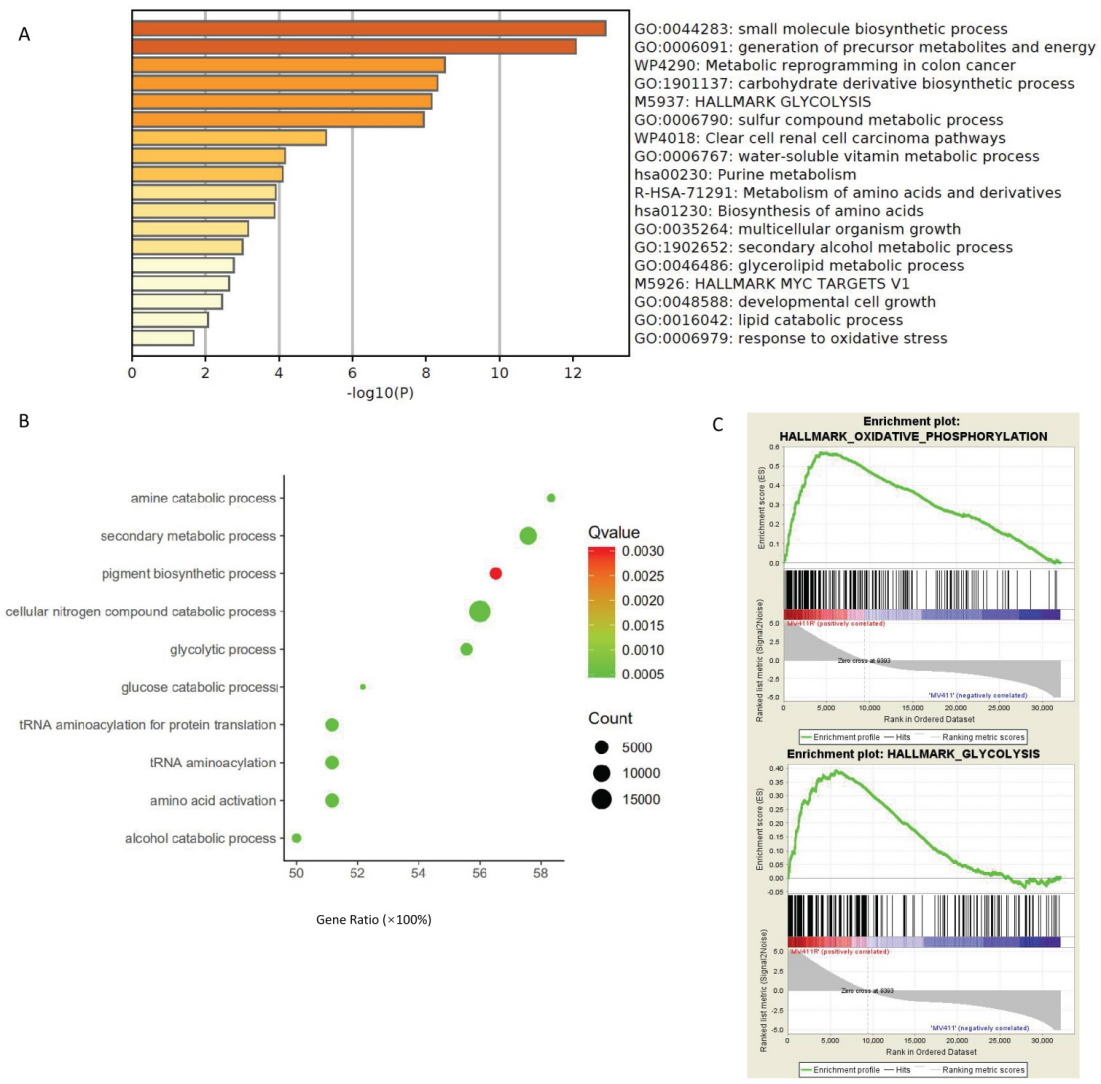
** Data mining to explore the relationship of drug sensitivity and aerobic glycolysis. A.** Multiple pathway enrichment of MV411 cells in an AC220 selected sgRNA library screen (GSE105161). Deep sequencing data for the sgRNAs dropped out following AC220 treatment were collected and the targeted genes were identified. Next the multiple pathway enrichment including GO, KEGG, HALLMARK and others were run on Metascape website. B. Gene ontology enrichment revealed the glucose metabolic related pathways in FLT3-ITD^+^AML. The analyze generated from array express data from EBI-TABM-1029. C. GSEA assay shown the enrichment in both oxidative phosophorylation and glycolysis pathways in sorafinib-resistant MV411 cell line (GSE74666).

First, Gallipoli, et al. 30 have performed a genome-wide CRISPR/Cas9 screen using FLT3 inhibitor AC220 to select genes and pathways that would sensitize FLT3-ITD AML to FLT3-TKI treatment, which provide us a perfect source (GSE105161) to study the connection between drug response and aerobic glycolysis. The multiple enrichment analysis done by Metascape revealed that the aerobic glycolysis related signal pathways were enriched in the genes identified as critical for resistance to FLT3 inhibitor AC220 (Figure 1A). Further, RNA sequencing data revealed that when treated with AC220, multiple metabolism related pathways were down regulated in MV411 cells (Figure S1A), including glycolysis, TCA cycle and glutaminolysis (Figure S1B).

Not only in the cell line experiments, we also found out that glycolysis signaling was activated in FLT3-ITD^+^AML in the patient samples. We performed gene ontology for the data acquired from the EBI-TABM-1029 database and found glycolysis related pathways were enriched in patients with FLT3-ITD^+^ present (Figure 1B).

AC220 is not the only FLT3 inhibitors that have connection to glucose metabolism, the glycolysis pathways and oxidative phosphorylation pathways were also upregulated in sorafenib resistant MV411 cells (Figure 1C), the dataset for GSEA analysis were from GSE74666.

### Treatment to FLT3-ITD^+^AML affects glucose metabolism and energy generation

**Figure 2:**
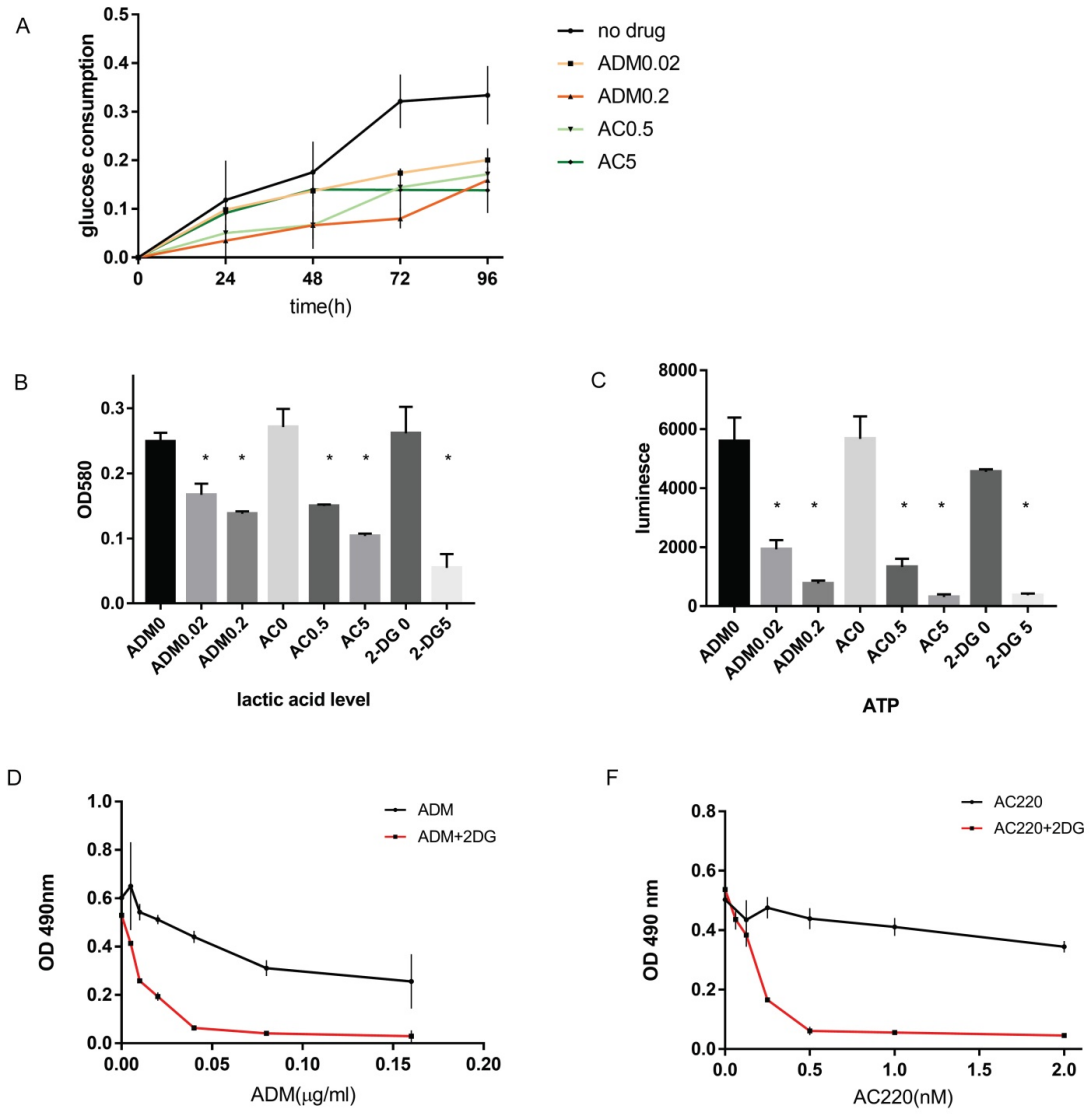
** Laboratory finding that validate the connection between drug sensitivity and glucose metabolism.** A. Time course of the glucose consumption for MV411 cells treated with different dose of ADM or AC220. The black line on the top is the glucose consumption of MV411 cells without treatment. Orange line and red lines stand for cells treated with 0.02 µg/mL and 0.2 µg/mL ADM; light green and dark green lines stand for cells treated with 0.5 µmol/L and 5 µmol/L AC220. B. Lactic acid generation of MV411 treated with different dose of ADM (0, 0.02 and 0.2 µg/mL), AC220 (0, 0.5 and 5 µmol/L) or 2-DG (0 and 5µmol/L). C. ATP level in various drug-treated MV411 cells. ADM0, ADM0.02, ADM0.2 represent the dose of ADM are 0, 0.02 and 0.2µg/ml; AC0, AC0.5, AC5 represent AC220 of 0, 0.5 and 5nM;2-DG0 and 2-DG 5 are the label of 2-DG 0 and 5mM. D& E. Dose-related growth inhibition by MTT assay for MV411 treat with ADM/AC220 with or without 2-DG.

Based on the bioinformatic findings, we performed *in vitro* experiments to determine how ADM and AC220 affects glucose metabolism and energy generation. Without drug treatment, about 30% of glucose consumption was observed and after 48 hours about 70% of glucose consumption was observed in MV411 cells. However, in MV411 cells treated with various concentration of ADM or AC220, the glucose residual in the culture medium increased and the glucose uptake decreased (Figure S2), and as the dose increased the consumption reduced (Figure 2A). The glucose consumption was both dose-dependent and time-dependent.

Since glucose in the media reduces significantly by 48 hours, we also determined lactic acid levels 48 hours post drug treatment. We chose two doses for treatment, the IC50 dosage and 10 times the IC50. Lactic acid generation was reduced upon AC220 or ADM treatment (Figure 2B), as was ATP generation (Figure 2C). When treated with 0.02 µg/mL ADM, lactic acid and ATP generation were reduced to 67% and 34.5%, respectively, of the levels found in the control cells. When the ADM dose was increased 10-fold, the lactic acid and ATP generation were further reduced to 55% and 13.7% of the control, respectively. When treated with 0.5 µM and 5 µM AC220, the lactic acid levels were reduced to 55% and 38%, and the ATP levels 23.4% and 5.5%, respectively, of the no treatment group. These results confirm that FLT3-ITD^+^AML inhibitors have function in adjusting glucose metabolism. Further supporting our hypothesis, blocking aerobic glycolysis by 2-DG synergized with chemotherapy or TKI inhibition *in vitro* (Figure 2D-E).

### miR-155 is a key factor in modulating drug resistance and aerobic glycolysis

**Figure 3:**
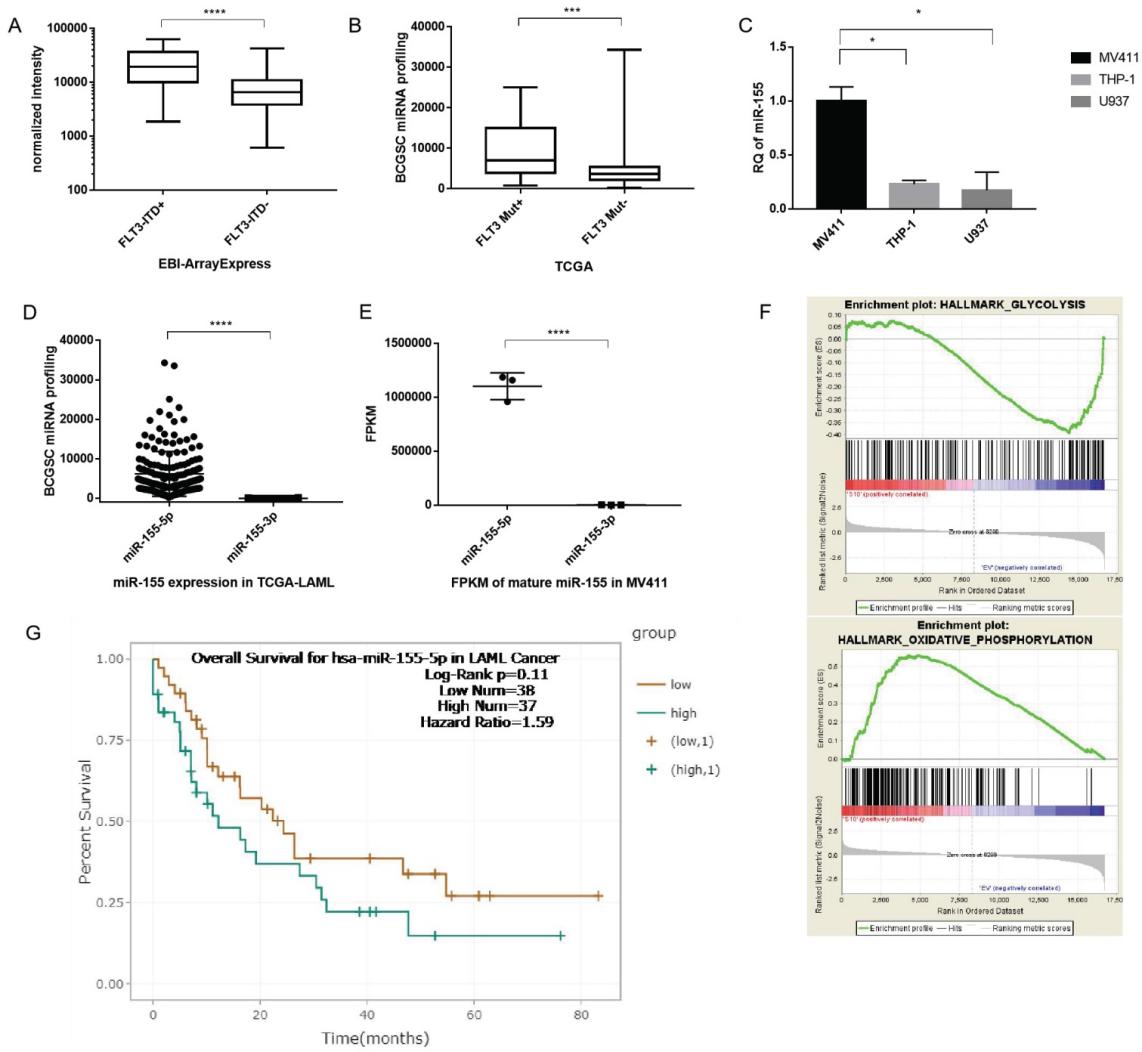
** Highly expressed miR-155 may have a positive role in modulating aerobic glycolysis in FLT3-ITD+AML.** A. Box plot of miR-155-5p expression between FLT3-ITD^+^AML and FLT3-ITD^-^AML. The expression level was quantified by normalized intensity. The array express data were acquired form dataset EBI-TABM-1032. B. Box plot of miR-155-5p expression between FLT3 mutation+ and FLT3 mutation- AML. The expression level was quantified by BCGSC profiling of miRNA. The miRNA sequence data were acquired form TCGA-LAML. C. miR-155-5p expression in MV411(FLT3-ITD^+^), THP-1 and U937 (both of these two were FLT3 wild type) cell lines. D. The expression level of miR-155-5p and miR-155-3p in TCGA-LAML dataset. E. The expression level of miR-155-5p and miR-155-3p in array express dataset. F. The mRNA expression file was generated from the RNA sequencing data of miR-155 knockdown MV411 cells were obtained from GSE71541, the GSEA analysis were done to identify the pathways involving aerobic glycolysis. G. Kaplan-Meier survival analysis were done to compare the overall survival of aberrant level of miR-155-5p, the figure is generated from the website ENCORI (http://starbase.sysu.edu.cn/ version 3.0).

After demonstrating that aerobic glycolysis activity is upregulated in FLT3-ITD^+^ cells and plays a key role in drug sensitivity, we investigated the signaling pathways involved in this modulation. First, we wanted to verify whether miR-155 was involved in the modulation. miR-155 is an abundant miRNA in AML and has been proven to have a role in activating FLT3 signaling. We analyzed the microarray data from EBI and found that compared to WT cells, the miR-155 level is significantly is upregulated in AML carrying the FLT3 mutation (Figure 3A). And when analyzing the data of AML from TCGA, we found that miR-155 expression higher in FLT3-ITD^+^AML than FLT3-ITD^-^ (Figure 3B). In the cultured cell lines, miR-155 was expressed 4 times higher in the FLT3-ITD^+^AML cell line MV411, than the FLT3 WT cell lines THP1 and U937 (Figure 3C).

Next, we evaluated which mature miRNA expressed in AML. The results showed miR-155-5p as the predominant miRNA in FLT3-ITD AML (with reads ranging from 225 to 34302), while reads of miR-155-3p were substantially lower (<205) (Figure 3D). We confirmed the low expression of miR-155-3p by sequencing MV411 cells (Figure 3E). Thus, we determined that miR-155-3p is not expressed in FLT3-ITD^+^AML and focused our further investigations on the role of miR-155-5p in FLT3-ITD^+^AML. Other factors supported miR-155-5p as the miRNA of interest, a higher miR-155-5p is corelated to a poorer overall survival (Figure 3G).

We then analyzed the RNA sequencing data of miR-155 from a GEO dataset (GSE71541). GSEA analysis showed the downregulation of the glycolysis and the activation of oxidative phosophoration pathways is correlated with the reduction in miR-155 (Figure 3F), which is indicating that miR-155 might have a positive role in modulating aerobic glycolysis. Thus, miR-155-5p is a highly expressed miRNA and may having a role in adjust glucose metabolism in FLT3-ITD+ AML.

### MiR-155 knockout impairs the cell viability in MV411 cells

**Figure 4:**
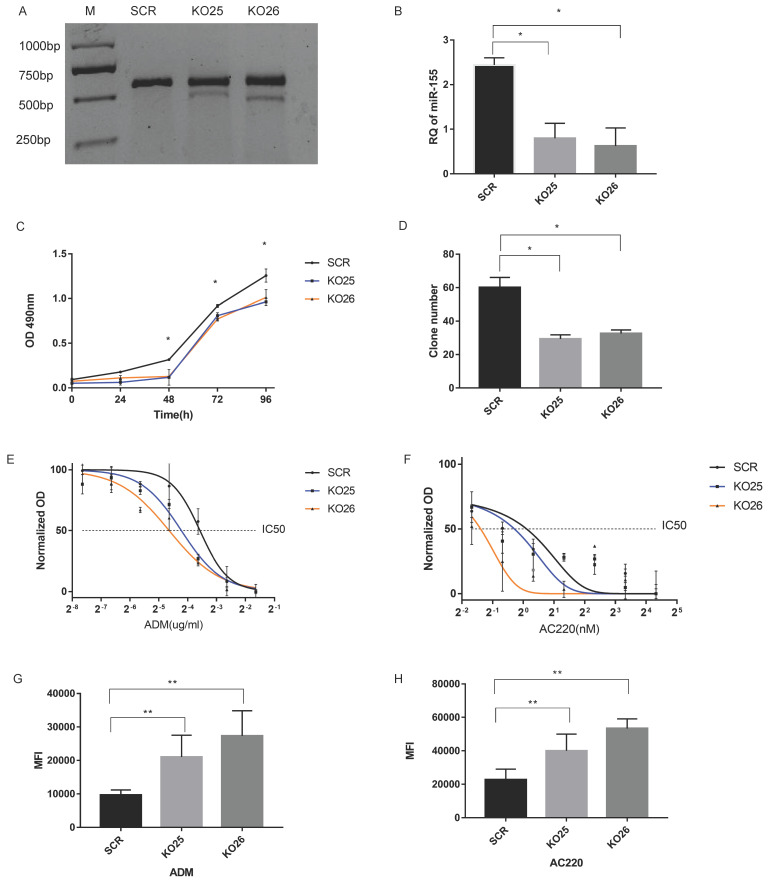
** Knockout of miR-155 in MV411 cells affect cell growth, drug resistance and drug accumulation.** A. Surveyor nuclease mutation assay for detection of the mutation by sgRNAs. The ratio of gray value between cleaved bands and total gray value (cleaved plus uncleaved) stands for the mutation rate. The mutation rate in KO25 were 15% and KO26 were 25%. B. Relative quantification (RQ) of miR-155-5p level by qPCR. C. Growth curve of MV411 with the 3 groups of miR-155 knockout pool in 24, 48,72 and 96 curves. D. Clone numbers of miR-155 knockout pools in the clone formation assay. E. Dose-related response in various concentration of ADM treatment ranging from 0 to 0.32 µg/ml with a 2-fold increase. Non-linear regression was done to estimate the relationship of dosage and treatment response. F. Dose-related response in various concentration of AC220 treatment ranging from 0 to 20 nM with a 4-fold increase. Non-linear regression was done to estimate the relationship of dosage and treatment response. G. Median fluorescence intensity (MFI) within cells treated with ADM were measured by flow cytometry. H. MFI within cells treated with AC220 and stained by Rhodamine123 fluorescent dye were measured by flow cytometry.

We generated miR-155 knockout MV411 cells via the CRISPR/Cas9 system and did both single sgRNA transduction (labeled as KO25 and KO26). Then we performed a surveyor assay to evaluate the mutation level in MV411 cells. The results in both KO25 and KO26 knockout clones were that the cleavage band and percentage of indel were at about 10% and 20%, respectively (Figure 4A). QPCR results revealed that there is a 70% decrease of miR-155 in KO25 cells and an 80% decrease in KO26 (Fi gure 4B). Therefore, both the sgRNAs can generate mutation in MV411 cells and successfully block mature miRNA expression. The cell growth curve revealed that cells with miR-155 knockout (KO25 and KO26) have a lower growth rate than SCR (Figure 4C). When compared the clone genic ability between miR-155 knockout cells and the control, the number of clones in KO25 and KO26 were only around 50% of SCR cells (Figure 4D) and the clones in miR-155-5p KO cells KO25 and KO26 were smaller in the size and contain less cells in one clone (Figure S3) when compared to SCR cells. We also did Wright's-Giemsa staining to observe the morphology change after miR-155 knockout, however, we fail to find out any significant morphology difference between the knockout and wild type (Figure S4). Thus, loss of miR-155 impairs the cell growth ability in MV411 cells.

### miR-155 levels are inversely correlated with sensitivity to Adriamycin and AC220

We next designed several experiments to analyze the relationship between miR-155 and drug sensitivity. First, we analyzed the dose-dependent response of MV411 cells to ADM and AC220, the relationship was plotted using a non-linear regression and calculate the IC_50_ of both drugs. The IC_50_ of each group are shown in Table 1 and Figure 3. The knockout of miR-155 increased both sensitivity to ADM (Figure 4E) and AC220 (Figure 4F) in MV411 cells.

Next, we used flow cytometry to assess drug accumulation in MV411 cells. Since ADM has a natural fluorophore moiety (emission wavelength ranging from 488 nm to 560 nm), we directly measured the florescence from ADM (Figure 4G). For AC220, we conducted a classic rhodamine-123 tracer dye experiment and again observed that miR-155 knockout resulted in increased cellular accumulation of the tracer dye (Figure 4H). Both the ADM and the rhodamine-123 results showed that miR-155 knockout cells have a stronger florescence signal, while the control without a decrease in miR-155 had less florescence, further supporting the important role miR-155 has in drug resistance.

To add adequate controls, we also knockout miR-155 in two other cell lines, the NB4 cells (a FLT3 WT AML cell line) and HeLa cell line (Cervical Cancer-Solid tumor) and conduct MTT assay to test the sensitivity to ADM. There is no difference between miR-155 knockout and control in NB4 cells (Figure S5A). The same trend was also observed in HeLa cells (Figure S5B). Only in MV411 cells, knockout of miR-155 increased the drug sensitivity to ADM and AC220.

### miR-155 positively regulates aerobic glycolysis

**Figure 5:**
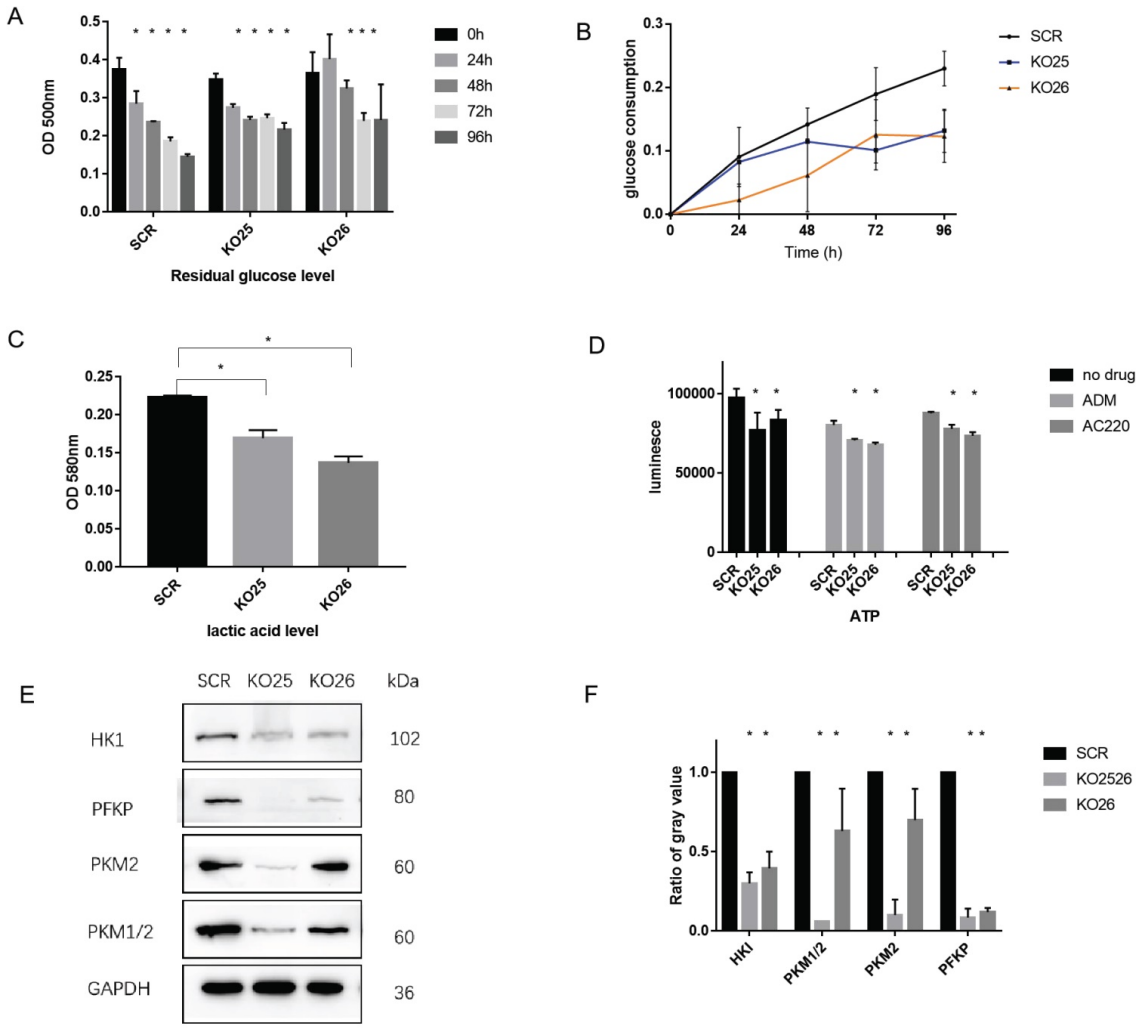
** miR-155 knockout inactivates aerobic glycolysis procedure in MV411 cells.** A. Glucose residual in miR-155 knockout cells. B. Glucose consumption in miR-155 knockout cells. C. Intracellular lactic acid level in miR-155 knockout cells. D. ATP level in the miR-155 knockout cells with or without treatment. E. Protein expression of glycolysis related enzyme in miR-155 knockout cells. F. Quantification of each protein expression by measuring the gray value.

To further validate whether changing the miR-155 level affects the extent of aerobic glycolysis, we performed a series of experiments. Downregulation of miR-155 demonstrated increased levels of glucose residuals (Figure 5A) and the glucose consumption decreased (Figure 5B). We also observed a lower lactic acid level (Figure 5C) and ATP level (Figure 5D) in miR-155 knockout cells. Specifically, we assayed the expression of genes with known glycolysis-related functions, and we found that HKI, PKM, PKM1/2, PFKP have significantly lower protein levels in miR-155 knockout cells (Figure 5E, F). These results indicate the functional roles miR-155 has in glycolysis.

### Overexpression of miR-155-5p affects drug resistance and metabolism

**Figure 6:**
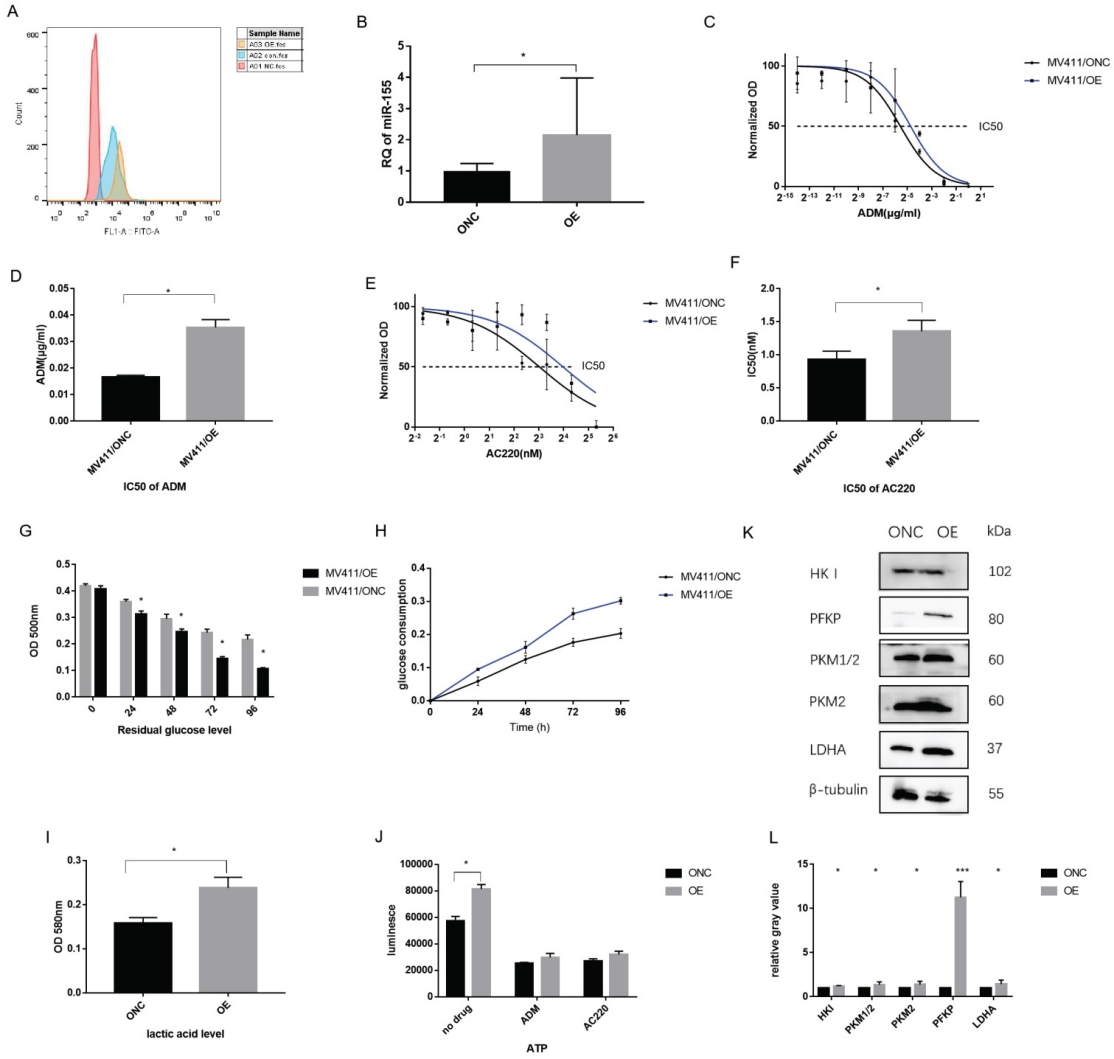
** Over-expression miR-155 in MV411 cells.** A. GFP expression in miR-155-5p transduced MV411 cells. B. miR-155 expression by QPCR in miR-155 overexpression cells and the control ONC. C. Dose-related response in various concentration of ADM treatment ranging from 0 to 1.0 µg/ml with a 4-fold increase. Non-linear regression was done to estimate the relationship of dosage and treatment response. D. IC50 of ADM in miR-155 OE MV411 cells. E. Dose-related response in various concentration of AC220 treatment ranging from 0 to 20 nM with a 4-fold increase. Non-linear regression was done to estimate the relationship of dosage and treatment response. F. IC50 of AC220 in miR-155 OE MV411 cells. G. Residual glucose in miR-155 OE and ONC groups of MV411 cells. H. The time-course of glucose consumption in MV411 cells. I. Lactic acid level in miR-155 OE and ONC cells. J. ATP production in miR-155 and ONC cells. K. Expression of glycolysis related protein in miR-155 OE and ONC cells. L. Quantification of gray value of each protein of glycolysis.

Inversely, we investigated the effect of overexpressing miR-155-5p in FLT3-ITD^+^AML. Over-expression of miR-155 was generated by a lenti-vector miR-155-5p sequence. After transduction, flow cytometry showed a GFP rate of about 95% (Figure 6A), indicating a high transduction rate further supported by qPCR, which showed a 4-fold upregulation of miR-155 (Figure 6B). A 2-fold increase in the IC_50_ of ADM and 1.5-fold increase in the IC_50_ of AC220 in miR-155 OE cells were observed (Table 2 and Figure 6C-F). In the miR-155 OE group, increased glucose consumption (Figure 6G, H) and lactic acid and ATP production (Figure 6 I, J) were observed as well as the opposite expression trends of those observed in the miR-155 KO cells. The increasing expression trends for glycolysis-related genes were observed in the miR-155 OE cells (Figure 6 K, L). Thus, we found that miR-155 overexpression has a role in promoting drug resistance and aerobic glycolysis.

### PIK3R1 is a direct target of miR-155

To identify the target of miR-155, we performed target prediction from starBase (http://starbase.sysu.edu.cn/) and found a list of potential mRNA targets 31, 32. Then we validate those targets by evaluating their expression level via sequencing data and qPCR results. Based on these results and cross referencing with the literature, we determined PIK3R1, a reported target in breast cancer, acts as a direct target of miR-155 in FLT3-ITD^+^AML.

**Figure 7:**
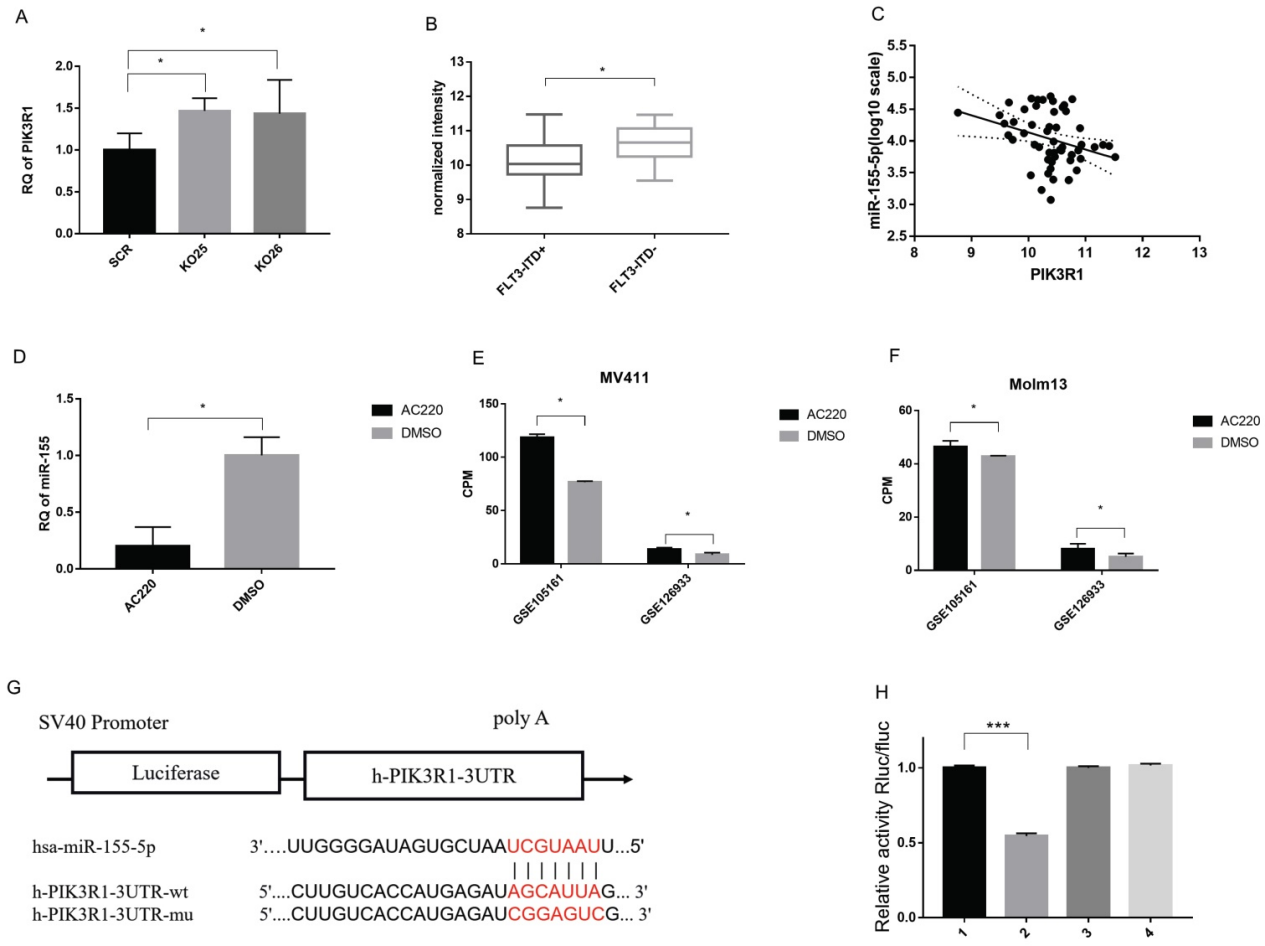
** PIK3R1 is a direct target of miR-155-5p.** A. RNA level of PIK3R1 in miR-155 KO and SCR cells. B. Negative co-expression relationship of PIK3R1 and miR-155-5p in Arrayexpress (see methods and materials). C. Expression level of PIK3R1 in FLT3-ITD+AML and FLT3-ITD-AML. D. Expression of miR-155 in AC220 treated MV411 cells. E&F. Expression of PIK3R1 in AC220 treatment of MV411 and Molm 13 cells. G. WT or MUT sequence of 3'UTR of PIK3R1 and miR-155-5p binding site. H. Luciferase activity in PIK3R1 WT and MUT cells in 293T cells and MV411 cells. Each lane stands for one group: 1. NCmimics+h-PIK3R1-3UTR-wt, 2. hsa-miR-155-5p+ h-PIK3R1-3UTR-wt, 3. NCmimics+h-PIK3R1-3UTR-mu, 4. hsa-miR-155-5p + h-PIK3R1-3UTR-mu.

We have multiple evidence to show the inverse trend of miR-155 and PIK3R1. Firstly, PIK3R1 was upregulated is miR-155 KO cells (Figure 7A). Second, the PIK3R1 level was significantly lower in FLT3-ITD^+^AML patients with the higher miR-155-5p level compared to FLT3-ITD^-^AML (Figure 7B), and the expression of miR-155-5p and the PIK3R1 were negatively correlated (Figure 7C). While miR-155 is downregulated upon AC220 and ADM treatment, qPCR shows that AC220 treatment upregulates the expression of PIK3R1 (Figure 7D). This trend is consistent in the sequencing data in MV411 cells and Molm13 cells (Figure 7E, F).

To prove miR-155-5p directly binds to the 3'-UTR, we mutated 3'UTR of PIK3R1 and perform a luciferase reporter assay in MV411 cells (Figure 7G). The results showed that luciferase signal is much lower in WT 3'UTR than other two mutants (Figure 7H), indicating a direct binding of miR-155-5p and the 3'UTR of PIK3R1. Thus, we considered PIK3R1 is a direct target of miR-155-5p.

**Figure 8:**
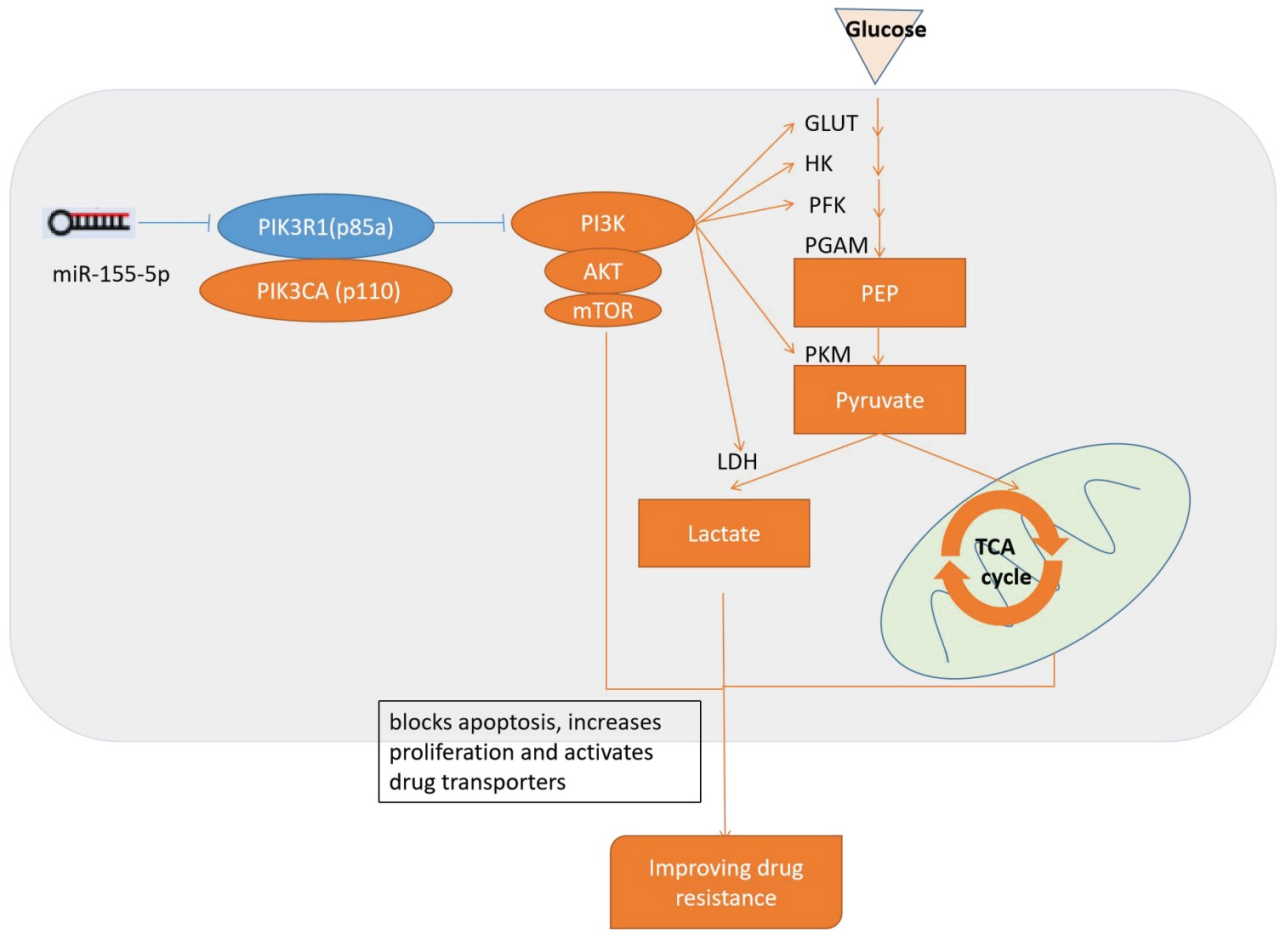
** Schematic diagram of the miR-155 regulates drug resistance.** miR-155-5p inhibits PIK3R1 and activates PI3K/Akt/mTOR pathways, which upregulates aerobic glycolysis and blocks apoptosis, increases proliferation, activates drug transporters and finally results in the progression of drug resistance.

Since PIK3R1 is a known inhibitor of PI3K/Akt pathway, and PI3K/Akt pathway have role to adjust drug resistance and glycolysis, we consider the inhibition of PI3K/Akt pathway by PI3KR1 is the reason that miR-155 knockout MV411 cells impaired glycolysis and thus overcome the treatment resistance in FLT3-ITD+AML (Figure 8).

## Discussion

As FLT3 mutations increase in AML and related carcinomas, TKIs have become a possible method of treating patients with FLT3-ITD mutations. Quizartinib (AC220) is a small-molecule TK1 receptor that can inhibit the class III receptor tyrosine kinases, such as FLT3 kinase. It has shown promise for treating patients with FLT3-ITD mutations. Due to patient resistance to drugs, however, the positive effects of FLT3 inhibitors are short-lived. This study determined that abnormalities in metabolism are a primary cause of patient resistance to certain drugs in FLT3-ITD^+^AML. Metabolic reprogramming is emblematic of resistance to drugs, with certain studies emphasizing how metabolites and metabolic enzymes can affect stem cell leukemogenesis and homeostasis 33. While enzymes have several direct effects such as energy production and consumption and biosynthesis, their indirect effects are equally important. These include regulating epigenetics, balancing redox, and regulating signal pathways. Metabolic adaptations can serve important roles in resistance processes when used during the treatment of hematological malignancies and solid cancers 34-37.

We also found that miR-155 significantly affected the modulation of metabolism as well as resistance to certain drugs. In FLT3-ITD^+^ AML, miR-155 is significantly upregulated compared with the WT FLT3 AML. The sensitivity of the drugs was increased by the knockout of miR-155, while overexpressing miR-155 raised its resistance to drugs. This shows that miR-155 is closely related to drug resistance. Higher miR-155 expression was associated with higher glucose usage and increased production of lactic acid, resulting in upregulated glycolysis. These glycolysis levels are significantly correlated with resistance to drugs while pausing glycolysis in miR-155 OE cells facilitated a more robust chemotherapeutic reaction against leukemia.

In AML, miR-155 is a mutated miRNA. The ectopic miR-155 expression found in hematopoietic progenitors can result in B-cell leukemia or a myeloproliferative disorder in mice, lending credence to the role of miR-155 as a leukemogenic factor 38. Increased miR-155 expression is an independent assessment factor of negative outcomes in typical patients, and is related to high-risk FLT3 ITD mutations 39-41. Downstream miR-155 marks that are associated with both clinical and typical hematopoiesis include PU.1^42^, which is a transcription factor essential for myelomonocytic differentiation, and the Src homology-2 domain-containing inositol 5-phosphatase 1 (SHIP1) tumor suppressor 43. The downregulation of these marks, which is mediated by miR-155, could be the cause of the oncomiR's leukemogenic capabilities.

PIK3R1 has recently been determined to suppress solid tumors 44-46. In this study, we found that PIK3R1 is a possible miR-155 target and that it is related to inhibiting drug resistance. PIK3R1 is the primary isoform regulating the tumor-suppressor gene phosphatidylinositol-4,5-bisphosphate 3-kinase (PI3K). In particular, PIK3R1/p85a is the most common isoform found in typical tissues, but displays reduced expression in cancerous tissues 47. The p85a is stabilized further by strongly attaching itself to the p110 catalytic subunit, inhibiting its catalytic activity. As the SH2 domains bind to activated RTK, it regulates PI3K's translocation to the plasma membrane and induces changes that contribute to its activation 48. Several genes activate PI3K, which results in protein kinase B (Akt) attaching to the cell membrane along with phosphoinositide-dependent kinase found in the PI3K/Akt signal transduction pathway. Previous research has demonstrated that the PI3K signaling pathways play a role in several cellular processes including motility, inflammation, metabolism, cell survival, and cancer progression 49. Additionally, the PI3K pathway can mediate the uptake of glucose 50-53. AKT assists in PI3K signaling, which mediates glucose transporter expression (GLUT1) and increases the capture of glucose while inducing activities related to phosphofructokinase. In these cases, activation of the PI3K pathways induces cell dependence on increased glucose levels 24, 54. One other possible outcome happens by way of the hypoxia-inducible factor (HIF)-1^55^. HIF-1α is associated with the severe hypoxic response related to erythropoietin, while HIF-2α is related to chronic hypoxia response 56. PI3K/Akt can also lower glycogen synthesis and raise glycolysis 57. While miR-155 can also raise HIF-1α expression to directly target miR-155, we did not see any HIF-1α increases in the miR-155 knockout cell lines or other indications that FLT3-ITD^+^AML cells target HIF-1α (unpublished data). Inhibiting PIK3R1 inactivates the PI3K/Akt signal, which is likely the reason that the metabolism is blocked, leading to increased drug resistance.

## Conclusion

MiR-155 is a key factor in modulating drug resistance and aerobic glycolysis in FLT3-ITD+AML. Our results highlight the importance of miR-155 and its downstream signaling in the control of leukemia cell metabolism. Extend our understanding of the role of metabolic adaptations in the resistance to TKIs treatment of FLT3. And provide an example, to be used as a template, for the design of novel and specific therapeutic strategies targeting cell metabolism in AML.

## Supplementary Material

Supplementary figures.

## Figures and Tables

**Table 1:** IC50 of ADM and AC220 in miR-155 knockout MV411 cells

IC50	SCR	KO25	KO26
ADM (µg/mL)	0.066±0.012	0.042±0.006	0.031±0.004
AC220(µM)	0.825±0.135	0.59±0.115	0.3±0.072

**Table 2:** IC50 of ADM and AC220 in miR-155 overexpressed MV411 cells

IC50	ONC	OE
ADM (µg/ml)	0.016±0.004	0.03±0.001
AC220(µM)	0.931±0.07	1.356±0.096

## Abbreviations

FLT3-ITD: Fms-like tyrosine kinase 3-internal tandem duplication
TKI: Tyrosine kinase inhibitors
miR-155: microRNA-155
CRISPR/Cas9: Clustered Regularly Interspaced Short Palindromic Repeats and CRISPR-associated (Cas) genes
MFI: median fluorescence intensity
DCFH: 2′, 7′-dichlorodihydrofluorescein
GEO: Gene Expression Omnibus
TCGA: The Cancer Genome Atlas
EBI: European Bioinformatics Institute
ENCORI: Encyclopedia of RNA Interactomes
GO: Gene ontology
KEGG: Kyoto Encyclopedia of Genes and Genomes
GSEA: Gene Set Enrichment Analysis
SEM: standard error of mean
