# Supplementary material for: Loss of MiR-155 Sensitizes FLT3-ITD+AML to Chemotherapy and FLT3 Inhibitors via Glycolysis Blocking by Targeting PIK3R1
Source: J Cancer. 2023 Jan 1;14(1):99–113. doi: 10.7150/jca.54775 (PMC9809327; doi:10.7150/jca.54775)
Supplement: Supplementary file 1 — Supplementary figures. [file jcav14p0099s1.pdf]

Figure S1

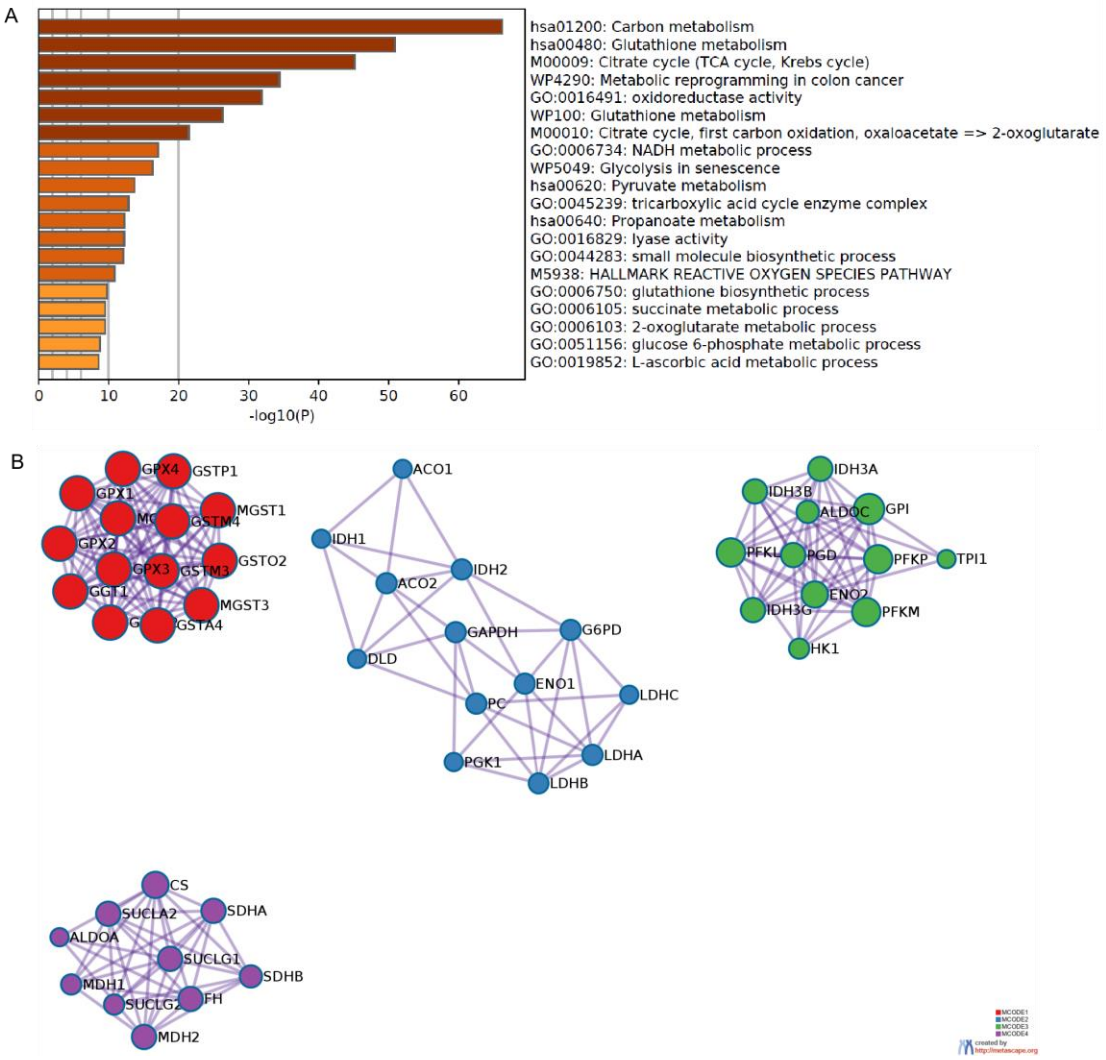

FigureS1. Glucose metabolism related genes in the MV411 cells treated with AC220.

A. Glucose metabolism related genes were filtered and did the multiple gene enrichment.

B.Clusters related in glucose metabolism for the MV411 cells treated with AC220. The protein-protein interaction network (PPI) using MCODE module in Cytoscape software 3.7.0. Red: Glutathione metabolism genes. Blue: TCA cycle. Green and purple: Glycolysis genes.

Figure S2

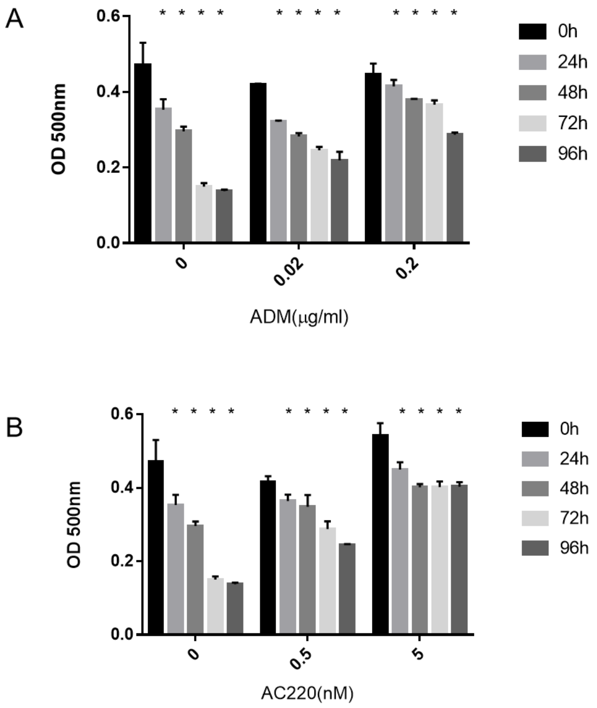

Figure S2.

A.The relative residual glucose level in the media of MV411 cell culture with ADM treatment of 0,0.02 and 0.2μg/ml respectively.

B.The relative residual glucose level in the media of MV411 cell culture with AC220 treatment of 0,0.5 and 5mM respectively.

FigureS3

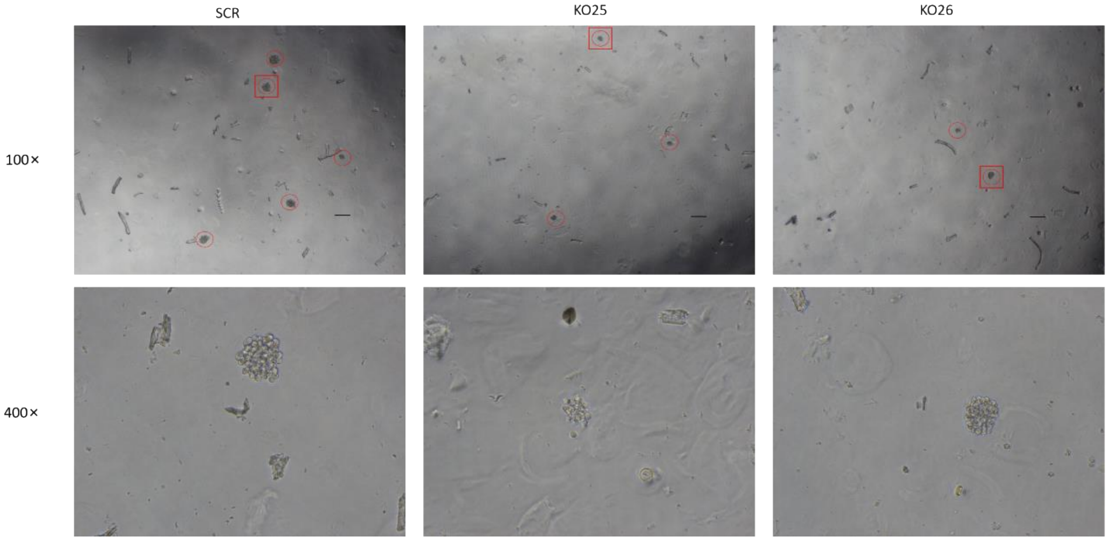

Figure S3. Cloney formation assay in methylcellulose. Two hundred cells were plated in media with 0.8 g/L methylcelluloses in each well on the 24-well plates and go on with regular cell culture. Fourteen days later, the number of colonies was counted under a phase-contrast microscope (Olympus) under the light field. From the left column to the right, the image was from SCR, KO 25, and KO26. The upper raw were images with 100× magnification, each red circle stands for one single cloney, and the bottom raw were the clones with better magnification (400×）within the red box above. Scale bar=100μm.

Figure S4

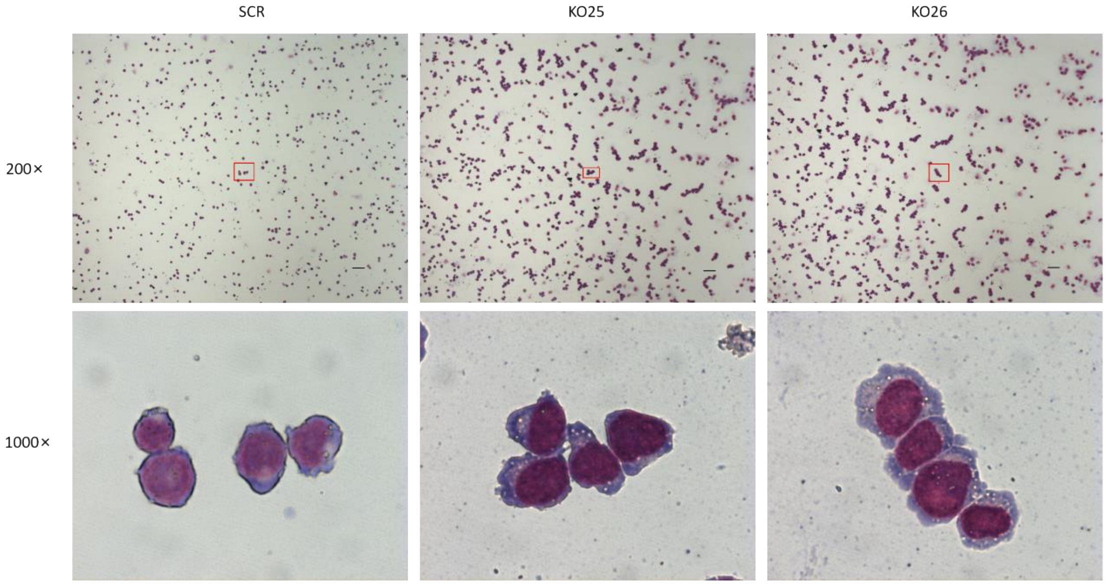

FigureS4. Cell morphology observation by Wright's-Giemsa staining of miR-155 KO cells. The cells were collected one week after transduction when the miR-155 knockout can be detected and cells have replicated about 2-3 generations. The same number of cells were collected, washed, and centrifuged to remove the supernatant then we made the cell smear and did the Wright's-Giemsa staining. The stained smear was imaged using an optical microscope (Olympus) under the light field. From the left column to the right, the image was from SCR, KO 25, and KO26. The upper raw is the image with 200 times magnification. And the bottom raw is a further magnification of the red boxes within the 200× image above, which is 1000× under the oil objective. Scale bar=50μm.

Figure S5

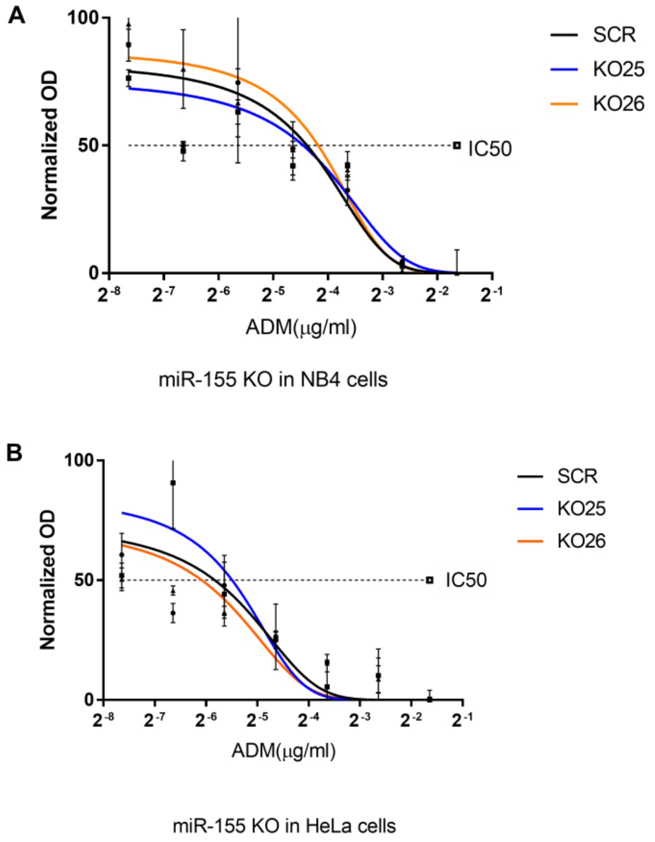

Figure S5. The dose-related inhibition of ADM in cells without FLT3-ITD expression. A. Knockout of miR-155 in NB4 cells. B. Knockout of miR-155 in HeLa cells.
